# Cyclotides Isolated from an Ipecac Root Extract Antagonize the Corticotropin Releasing Factor Type 1 Receptor

**DOI:** 10.3389/fphar.2017.00616

**Published:** 2017-09-25

**Authors:** Mohsen Fahradpour, Peter Keov, Carlotta Tognola, Estela Perez-Santamarina, Peter J. McCormick, Alireza Ghassempour, Christian W. Gruber

**Affiliations:** ^1^Center for Physiology and Pharmacology, Medical University of Vienna Vienna, Austria; ^2^Medicinal Plants and Drugs Research Institute, Shahid Beheshti University Tehran, Iran; ^3^Faculty of Medicine, School of Biomedical Sciences, The University of Queensland, Brisbane QLD, Australia; ^4^School of Veterinary Medicine, University of Surrey Guildford, United Kingdom

**Keywords:** plant peptides, circular peptide, pharmacognosy, ipecac, GPCR, corticotropin-releasing factor

## Abstract

Cyclotides are plant derived, cystine-knot stabilized peptides characterized by their natural abundance, sequence variability and structural plasticity. They are abundantly expressed in Rubiaceae, Psychotrieae in particular. Previously the cyclotide kalata B7 was identified to modulate the human oxytocin and vasopressin G protein-coupled receptors (GPCRs), providing molecular validation of the plants’ uterotonic properties and further establishing cyclotides as valuable source for GPCR ligand design. In this study we screened a cyclotide extract derived from the root powder of the South American medicinal plant ipecac (*Carapichea ipecacuanha*) for its GPCR modulating activity of the corticotropin-releasing factor type 1 receptor (CRF_1_R). We identified and characterized seven novel cyclotides. One cyclotide, caripe 8, isolated from the most active fraction, was further analyzed and found to antagonize the CRF_1_R. A nanomolar concentration of this cyclotide (260 nM) reduced CRF potency by ∼4.5-fold. In contrast, caripe 8 did not inhibit forskolin-, or vasopressin-stimulated cAMP responses at the vasopressin V_2_ receptor, suggesting a CRF_1_R-specific mode-of-action. These results in conjunction with our previous findings establish cyclotides as modulators of both classes A and B GPCRs. Given the diversity of cyclotides, our data point to other cyclotide-GPCR interactions as potentially important sources of drug-like molecules.

## Introduction

Historically plants have been a rich source for drug discovery. For example salicylic acid, one of the most well-known antipyretic, anti-inflammatory and analgesic drugs, was originally derived from willow bark (*Salix alba*) (Aronson, 2013). Similarly, the discovery of artemisinin, a highly effective compound against the Malaria parasite was first isolated from *Artemisia annua* (Tu, 2011), led to the award of the Nobel Prize in Physiology and Medicine in 2015 (Efferth et al., 2015). Such discoveries have commonly involved small molecules. However, plant peptides are gaining consideration for new opportunities in drug discovery and development (Craik et al., 2013).

Plants produce a variety of peptides that comprise hormones for cellular signaling, secretory peptides for interspecies communication, and defense peptides against microbes, herbivores and pests (Marmiroli and Maestri, 2014). An interesting group of plant-derived peptides with applications in drug discovery are cyclotides, which belong to the large class of ribosomally synthesized and post-translationally modified peptides (Arnison et al., 2013). Cyclotides are disulfide-rich peptides (∼30 amino acids) that contain a head-to-tail cyclized backbone and six conserved cysteine residues forming three knotted disulfide bonds. This unique topology, known as the cyclic cystine-knot motif (Craik et al., 1999), confers them a tightly packed three-dimensional fold and makes them notably stable against thermal, chemical, and enzymatic degradation (Colgrave and Craik, 2004). In addition, cyclotides exhibit an unprecedented variability and natural abundance: a single species can express over 150 different cyclotides (Hellinger et al., 2015b; Serra et al., 2016). In total, the number of unique cyclotides to be discovered in plants has been estimated to exceed 100,000 (Hellinger et al., 2015b), hence, making this group of peptides one of the most abundant and diverse of plant origin (Gruber et al., 2008; Burman et al., 2015). Moreover, due to their stability and structural plasticity (Clark et al., 2006), cyclotides have attracted attention as potential frameworks for peptide-based drug design and pharmaceutical applications (Craik and Du, 2017).

Besides the natural role of cyclotides as plant defense agents (Gruber et al., 2007; Craik, 2012), they exhibit a broad range of pharmaceutically relevant activities including cytotoxicity (Burman et al., 2011), uterotonic (Gran et al., 2008; Gruber and O’Brien, 2011), anticancer (Esmaeili et al., 2016), anti-HIV (Wang et al., 2007) and immunosuppressive properties (Gründemann et al., 2012, 2013; Hellinger et al., 2014; Thell et al., 2016). Furthermore, together with the discovery of a cyclotide agonist of the human oxytocin and vasopressin 1a receptors, cyclotides can be utilized as natural templates for G protein-coupled receptor (GPCR) ligand design (Koehbach et al., 2013b).

Cyclotides have been isolated and characterized from different flowering plant species of the Rubiaceae, Violaceae, Cucurbitaceae, Fabaceae, Solanaceae, and Poaceae families [summarized in (Hellinger et al., 2015b; Koehbach and Clark, 2016)]. Inspired by their original discovery from *Oldenlandia affinis*, the Rubiaceae family, has been a major focus for studying the diversity and distribution of cyclotides (Gruber et al., 2008; Gruber, 2010; Koehbach et al., 2013a). In particular the genus Psychotria *sensu lato* (*s.l.*) provides enormous resource for the discovery of pharmacologically active cyclotides. For example, purification of the cyclotide cyclopsychotride A from an extract of *Psychotria longipes* (now reclassified as *P. vellosiana*), identified a blocker of neurotensin 1 receptor signaling (Witherup et al., 1994), another representative of the GPCR family. Previously we discovered cyclotides in *P. ipecacuanha* (*Brot.*) [=*Carapichea ipecacuanha* (*Brot.*)] using an innovative transcriptome-mining approach (Koehbach et al., 2013a). A herbal preparation of the root extract of *C. ipecacuanha* is commonly known as ‘syrup of ipecac,’ which has a long history in traditional medicine and has been used in Western clinical practice until the late 20th century for its properties as emetic and expectorant agent. It is well documented that the major alkaloids emetine and cephaeline are causing these effects (Lee, 2008). However, hitherto there are no reports of pharmacological activities of cyclotides derived from the root extract of ipecac.

Knowing that cyclotides are able to modulate GPCR signaling of representative class A receptors [i.e., the oxytocin- (Koehbach et al., 2013b), vasopressin V_1a_- (Koehbach et al., 2013b) and neurotensin 1 receptors (Witherup et al., 1994)] and that they are abundantly expressed in plants of the genus Psychotria, the present study aimed to expand our exploration of cyclotides as ligands of other GPCRs. In particular, we focused on identifying novel cyclotides present in *C. ipecacuanha* and investigation of their properties to modulate signaling of the corticotropin-releasing factor type 1 receptor (CRF_1_R) using a bioassay-guided fractionation approach combined with pharmacological and structural analysis. The CRF_1_R is a prototypical class B GPCR, and together with its endogenous ligand CRF (a peptide hormone containing 41 amino acids), regulates the hypothalamic-pituitary-adrenal axis that provokes cortisol release and coordinates the endocrine response to stress behaviors in the central nervous system (Gutman et al., 2003). The CRF peptide and its receptor are also involved in the response of the gut to stress-related colonic functions. Hence CRF_1_R signaling is thought to be an important therapeutic target of gut-related stress disorders, such as irritable bowel syndrome (Tache et al., 2004), as well as to treat anxiety, depression and drug addiction (Zorrilla and Koob, 2010; Logrip et al., 2011; Navarro et al., 2015).

Here, we analyzed a cyclotide-rich root extract of *C. ipecacuanha* by reversed-phase high-performance liquid chromatography (RP-HPLC) and matrix-assisted laser desorption ionization time-of-flight (MALDI-TOF) mass spectrometry (MS) utilizing chemical derivatization and enzymatic digest. Using a robust, cell-based luciferase reporter assay we isolated novel cyclotides that antagonized the CRF_1_R, which has been confirmed by quantitative second messenger analysis. The structures of these cyclotides were elucidated by combining *de novo* peptide sequencing and transcriptome analysis. This is to our knowledge the first report demonstrating cyclotides as modulators of class B GPCR signaling and further advances our understanding of their diverse molecular mechanism of action.

## Results

### Preparation and Analysis of Cyclotide-Containing Plant Extracts

**FIGURE 1:**
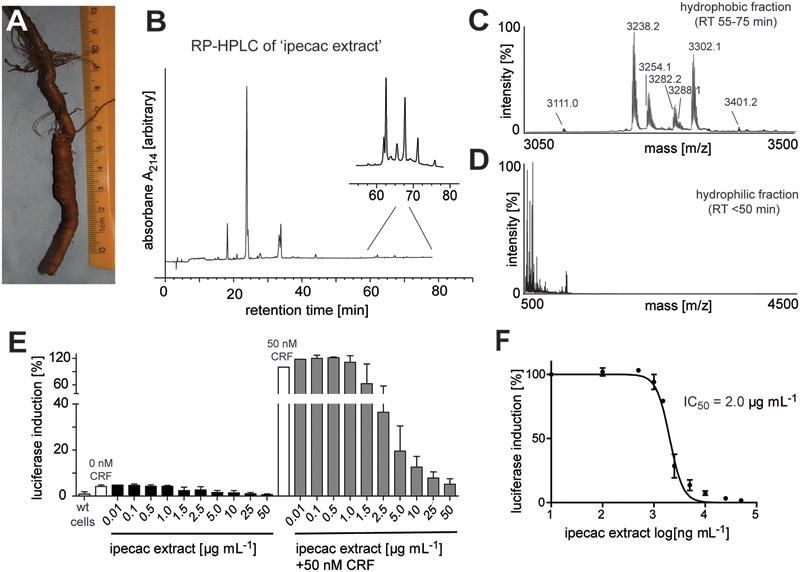
Analytical profile of ipecac cyclotide extract. **(A)** Image of a representative *Carapichea ipecacuanha* root [photograph by courtesy and with permission of Orlando Vargas Ramírez (Vargas, 2017), Organization for Tropical Studies, Costa Rica]. **(B)** Analytical reversed-phase liquid chromatography (RP-HPLC) of crude cyclotide-enriched extract of *C. ipecacuanha*, with magnification of cyclotide fractions (inset), performed using linear gradients as described in the section “Materials and Methods.” **(C,D)** Matrix-assisted laser desorption ionization time-of-flight (MALDI-TOF) mass spectra of **(C)** the hydrophobic cyclotide-rich fraction and **(D)** the hydrophilic non-cyclotide containing fraction from the crude extract. **(E)** Inhibition of human CRF-induced CRE-specific luciferase activity by ipecac cyclotide extracts. The data presented are mean ± SD of five separate experiments performed in triplicate. **(F)** The ipecac extract inhibits human CRF response in a concentration-dependent manner with an IC_50_ of 2.0 μg mL^-1^. Masses labeled in the spectra refer to monoisotopic [M+H]^+^.

Based on previous methods for cyclotide extraction and identification (Hashempour et al., 2013; Koehbach et al., 2013a; Hellinger et al., 2015a), we isolated and characterized cyclotides from ipecac root (**Figure 1A**) powder (*C. ipecacuanha*) with the aims to expand our knowledge of cyclotide sequence diversity and to investigate their modulating effects on signaling of class B GPCRs. The initial aqueous root extract of ipecac was prepared by maceration; this extract was then pre-purified by C_18_ solid-phase extraction to remove polar plant constituents. This resulting ‘ipecac extract’ was analyzed by RP-HPLC to confirm the presence of absorbance signals across a linear acetonitrile gradient (**Figure 1B**). Due to the hydrophobic surface properties of cyclotides, they elute late in reversed-phase chromatography. Hence we manually collected the early- (hydrophilic) and late-eluting (hydrophobic) fractions to analyze the presence of cyclotides in the ipecac extract via MALDI-TOF MS. As expected, mass signals characteristic for the presence of cyclotides were identified within the molecular weight range of 2500–4000 Da (**Figure 1C**), but were absent on the more hydrophilic fraction of the extract (**Figure 1D**). Given our interest in the discovery of novel GPCR-modulating compounds, we measured effects of the ipecac extract on CRF_1_ receptor signaling.

### Modulating Effect of Ipecac Extract on CRF_1_ Receptor Signaling

The ipecac extract was tested for its modulating effect on CRF_1_R signaling using a cell-based luciferase reporter assay. HEK293 cells transiently transfected with an C-terminally GFP-tagged human CRF_1_R and a luciferase-coupled cAMP response element (CRE) were stimulated with varying concentrations of ipecac extract (0.01–50 μg mL^-1^) in the presence and absence of 50 nM endogenous corticotropin-releasing factor (CRF) ligand. Concentration-dependent inhibition of receptor signaling was determined by measuring luciferase induction and accounting for the fluorescence of receptor-positive cells. The amount of luminescence of the control samples incubated with hormone alone was defined as 100% efficacy (**Figure 1E**). The inhibitory potency of the corresponding cyclotide-containing ipecac extract was measured and normalized relative to the efficacy of the control. The ipecac extract exhibited CRF-stimulated CRF_1_R inhibition in a concentration-dependent manner, whereas the extract only did not induce any effect (**Figure 1E**). The inhibitory potency of the ipecac extract to antagonize CRF_1_R signaling was determined as half-maximal concentration (IC_50_) as 2.0 μg mL^-1^ (**Figure 1F**). Assuming that the ipecac extract contained cyclotides, it was reasonable to further determine the typical structural properties of these peptides, i.e., the presence of six cysteines and a circular peptide backbone.

### Identification of Cyclotides in the Cyclotide-Enriched Ipecac Fraction

**FIGURE 2:**
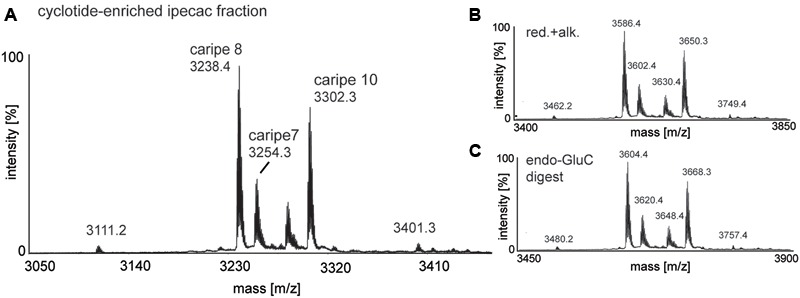
MALDI-TOF mass spectrometry (MS) of crude and chemically modified cyclotide-enriched extract. Mass spectra of **(A)** unmodified extract, **(B)** reduced and alkylated crude extract (red.+alk.) using dithiothreitol and iodoacetamide, and **(C)** endo-GluC digested extract are shown. Masses labeled in the spectra refer to monoisotopic [M+H]^+^ and cyclotides are labeled according to **Table 1**. Using this analysis workflow, circular peptides containing six cysteines typically exhibit a mass shift of +348 Da (red.+alk.) and +366 Da (endo-GluC).

Given the antagonism of CRF_1_R-mediated luciferase activity by the ipecac extract, we sought to identify the cyclotides present in the extract. The ipecac extract was subject to bio-chemical derivatization by thiol-reduction, carbamidomethylation and endopeptidase treatment, using dithiothreitol, iodoacetamide, and endoproteinase (endo-)GluC, respectively. Cyclotides generally, and particularly caripe cyclotides, contain a single conserved glutamic acid residue (Koehbach et al., 2013a). Hence, digestion with endo-GluC is a valuable tool for analyzing cyclotides. It was clear that the mass signals (e.g., 3254.3, 3238.4, and 3302.3 Da) in the unmodified extract (**Figure 2A**) shifted by 348 ± 0.1 Da (i.e., 3602.4, 3586.4, and 3650.3 Da) after reduction and alkylation (**Figure 2B**), characteristic of peptides containing six thiol-oxidized cysteine residues. In addition, the masses of these peptide signals increased by another 18 Da (i.e., 3620.4, 3604.4, and 3668.3 Da) after enzymatic digest with endo-GluC (**Figure 2C**), corresponding to the addition of a H_2_O molecule, typical for the enzymatic ring-opening of a backbone cyclized peptide.

### Bioactivity-Guided Fractionation of Cyclotide-Containing Ipecac Extract

**FIGURE 3:**
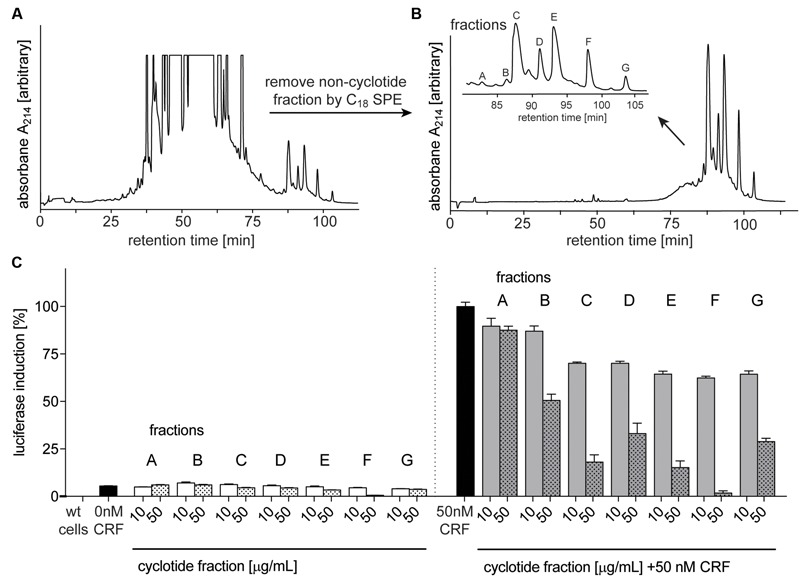
Purification and bioassay-guided fractionation of ipecac cyclotides. Preparative RP-HPLC A_214_ chromatograms of crude extract of *C. ipecacuanha* **(A)** before and **(B)** after cyclotide-enrichment via solid-phase extraction. (**B**, inset) shows the elution profile of the main cyclotide fractions A–G. **(C)** Inhibition of human CRF_1_R by cyclotide fractions (A–G), in the absence (left panel) and presence of the endogenous agonist, CRF, (50 nM; right panel). Data were normalized to the response of CRF (50 nM) alone. Cyclotides were dissolved in ddH_2_O for luciferase assays and tested at concentrations of 10 (solid bars) and 50 (dotted bars) μg mL^-1^ for their ability to inhibit CRF (black bars) signaling. Data shown are mean ± SD of two experiments.

**FIGURE 4:**
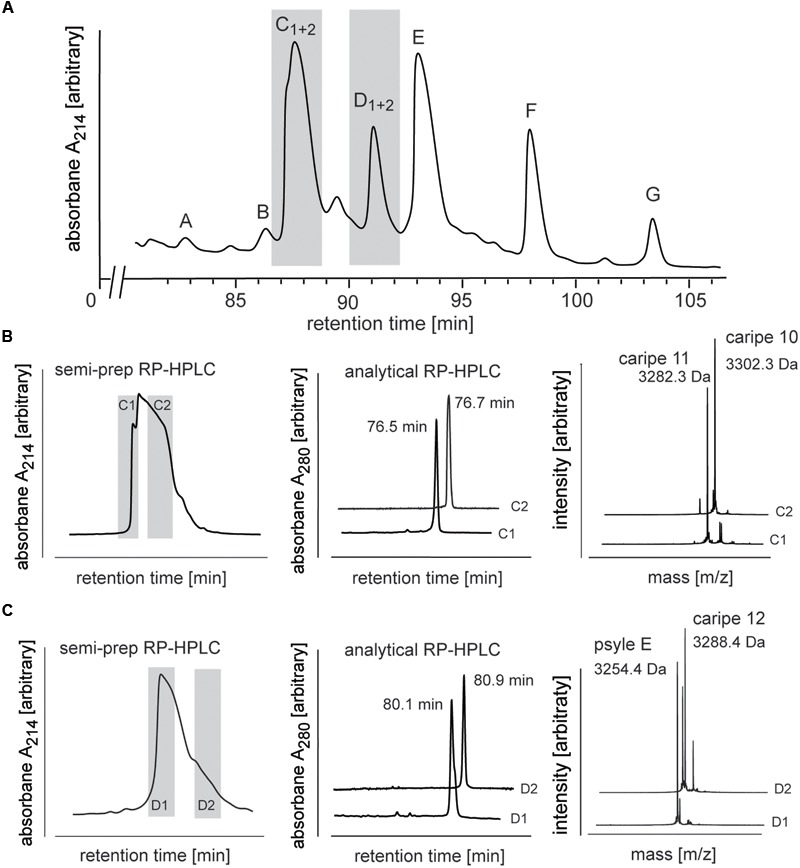
Analysis and purification of co-eluting Carapichea cyclotides. **(A)** Preparative RP-HPLC of cyclotide-enriched fraction of *C. ipecacuanha* shown in the cyclotide-eluting region of the chromatogram. Cyclotide fractions A–G are indicated. Co-eluting cyclotides in fraction C and D were further analyzed and purified. **(B,C)** The two cyclotides in **(B)** fraction C (caripe 10 and caripe 11) and **(C)** fraction D (caripe 12 and psyle E) were purified by semi-preparative RP-HPLC (left panel) and characterized by analytical RP-HPLC (A_214_ trace indicating elution times) (middle panel) and MALDI-TOF MS (right panel). Masses labeled in the spectra refer to monoisotopic [M+H]^+^.

After confirming the presence of cyclotides in the ipecac extract, we aimed to isolate the cyclotides responsible for CRF_1_R antagonism using a bioassay-guided fraction approach. Despite the presence of cyclotide in the ipecac extract, the HPLC chromatogram of the ipecac extract (**Figure 3A**) indicated the presence of early-eluting, hydrophilic non-cyclotide compounds. To remove these unwanted impurities and for improvement of preparative cyclotide fractionation yield, the ipecac extract was pre-fractionated by solid-phase extraction as explained in the section “Materials and Methods” (**Figures 3A,B**). The resulting cyclotide-enriched ipecac fraction was subject to fractionation by preparative HPLC, and manual collection of seven cyclotide fractions as indicated in alphabetical order (A–G) in the chromatograms (**Figure 3B**). All fractions were analyzed by analytical HPLC and MALDI-TOF MS. Fractions A and B only contained trace amounts of cyclotides, whereas fractions E, F, and G each contained individual cyclotides with high purity (Supplementary Figure 1). Fractions C and D were determined to contain at least two cyclotides (**Figures 4B,C** and Supplementary Figure 1). All fractions were examined with the luciferase activity assay, as described earlier, and tested for agonism/antagonism at the CRF_1_R. We tested two concentrations per fraction (10 and 50 μg mL^-1^) in the absence and presence of CRF ligand (**Figure 3C**). None of the fractions mediated any changes in luciferase signal when applied to the CRF_1_R cells alone. On the other hand all fractions A–G of the cyclotide-enriched ipecac fraction were capable of inhibiting the CRF-stimulated (50 nM) luciferase induction, suggesting that the cyclotides antagonize, but do not activate the CRF_1_R. Analyzing the percentage of inhibition, fraction F appeared to be the most effective antagonist, which inhibited CRF-induced luciferase signal by 37.6 and 98.2% at 10 and 50 μg mL^-1^, respectively. These results suggested that the ipecac extract, and the cyclotide-enriched fraction, respectively, contain cyclotides that inhibit the CRF_1_R with varying inhibitory potential. It was therefore of major importance to characterize these cyclotides at a molecular level, by further purification and amino acid sequencing.

### Purification of Co-eluting Cyclotides

Analytical RP-HPLC and MALDI-TOF MS analyses confirmed the purity of all fractions except C and D (Supplementary Figure 1). MALDI-TOF MS spectra of the latter two fractions indicated the presence of multiple mass signals corresponding to at least two cyclotides in each fraction. Since preparative scale RP-HPLC system was not able to discriminate and resolve these co-eluting peptides (**Figure 4A**), we utilized a semi-preparative method to isolate these ***Car****apichea* ***ipe****cacuanha* (caripe) cyclotides to purity. Fraction C eluted between 78–83 min and was collected as two sub-fractions C1 (78–79 min) and C2 (80–82 min) (**Figure 4B**). MALDI-TOF MS spectra and analytical HPLC analysis of each fraction confirmed their high purity, and indicated the presence of a single, distinct cyclotide in each fraction, caripe 10 and caripe 11, respectively (**Figure 4B** and Supplementary Figure 1). The semi-preparative HPLC chromatogram of fraction D contained a broad peak eluting between 83 and 87 min, which was collected as two sub-fractions D1 (83–84 min) and D2 (85–86 min) (**Figure 4C**). MALDI-TOF MS spectra and analytical HPLC revealed the separation of distinct HPLC peaks in fractions D1 and D2 that were later sequenced as psyle E and caripe 12, respectively (**Figure 4C** and Supplementary Figure 1).

### *De Novo* Sequencing of Caripe Cyclotides

**FIGURE 5:**
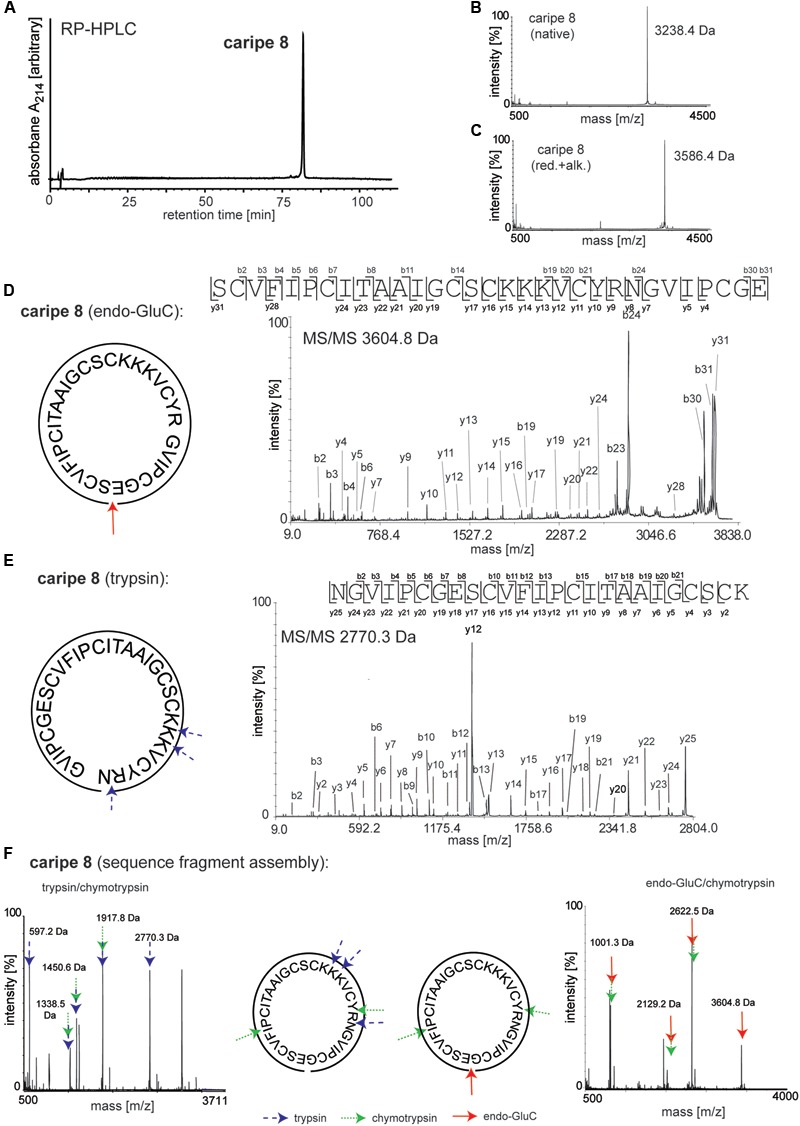
*De novo* sequencing of the cyclotide caripe 8. Purity and molecular weight of caripe 8 was analyzed by **(A)** analytical RP-HPLC and **(B)** MALDI-TOF MS. **(C)** The presence of the six cysteines, typical for cyclotides was confirmed via reduction and alkylation (red.+alk.) followed by MS. Cyclotide structure was elucidated by *de novo* peptide sequencing, comprising the analysis of a combination of single and multiple trypsin (blue arrows, dashed line), chymotrypsin (green arrows, dotted line) and endoproteinase GluC (endo-GluC; red arrows, solid line) digests. Tandem mass spectra of **(D)** an endo-GluC digest and **(E)** a trypsin digest are illustrated exemplarily. The amino acid sequence was determined by manual assignment of the N-terminal b-ion and C-terminal y-ion series and the ion fragmentation calculator tool (Data Explorer^TM^, AB Sciex). **(F)** The full sequence was determined via sequence fragment assembly by interpretation of digest fragments using combination of trypsin and chymotrypsin (left panel) and endo-GluC and chymotrypsin (right panel). Fragments of the different digests are indicated by arrows. Masses labeled in the spectra refer to monoisotopic [M+H]^+^.

Amino acid sequences of purified cyclotides derived from fractions C-G (**Figure 3B** and Supplementary Figure 1) were obtained using an optimized MALDI-based peptidomics approach, termed ‘sequence fragment assembly’ (Hashempour et al., 2013). The trace amounts of cyclotides in fractions A and B precluded their inclusion for sequencing. The overall workflow is illustrated for caripe 8 (**Figure 5**) and shown for other *de novo* sequenced cyclotides in the Supplementary Figures 2–7. First, the purified, native cyclotides (**Figures 5A,B**) were chemically modified to yield S-carbamidomethylation of cysteines. This included the reduction of disulfide bonds with dithiothreitol, and the alkylation of reduced sulfhydryl groups with iodoacetamide, which yielded a mass shift of 348.1 Da (**Figure 5C**), indicative of the presence of six cysteine residues (as mentioned previously). Afterward, the fully reduced and alkylated peptides were digested with a single enzyme, i.e., trypsin or endo-GluC to produce linear peptide chains amenable to fragmentation by MS/MS. The resulting spectra were analyzed manually by allocating N-terminal b- and C-terminal y-ions (**Figures 5D,E**). Due to the presence of only one conserved glutamic acid residue in the cyclotide sequence, an endo-GluC digest will usually provide a complete C-N ion series of the linearized precursor ion (caripe 8, 3604.8 Da). On the other hand, tryptic digests of cyclotides derived from HPLC fractionation often results in undistinguishable fragmentation patterns due to multiple enzyme cleavage sites (Arg and Lys), and hence multiple (and sometimes very small fragments, or single amino acids) (Hashempour et al., 2013) (for caripe 8 only the largest precursor with 2770.3 Da provided useful sequence information, **Figure 5E**). To overcome this problem in *de novo* cyclotide sequencing, single and double digests combining trypsin, endo-GluC and chymotrypsin were applied to generate smaller fragments with distinct molecular weight (**Figure 5F**). By combining the annotated sequence information derived from the molecular weight of fragments and alignment with the assigned endo-GluC ion series it was possible to assemble the full cyclotide sequence. In the example of caripe 8, the combination of trypsin/chymotrypsin (1338.5, 1450.6, and 1917.8 Da) and endo-GluC/chymotrypsin provided each three distinct fragments (1001.3, 2129.2, and 2622.5 Da) (**Figure 5F**). Each full length sequence was generated by alignment of the sequenced endo-GluC and tryptic ion series, with the annotated fragments of the trypsin/chymotrypsin and endo-GluC/chymotrypsin digest. Finally, sequences were confirmed by automated ion fragmentation analysis, chymotrypsin fragmentation pattern, sequence homology, and amino acid analysis (see Materials and Methods section). Applying this approach for sequencing of cyclotides isolated from ipecac fraction A–G led to the identification of seven cyclotides, four of which (caripe 10, 11, 12, and 13) were previously unknown sequences (**Table 1**). Of the three known cyclotides, two cyclotides, caripe 7 and 8, were only characterized at a transcriptome level by tBLASTn (Wang et al., 2008) mining of the 1kp dataset (Koehbach et al., 2013a), and their molecular structure was here for the first time confirmed at a peptide level (**Table 1**). The third cyclotide, psyle E has been previously isolated from *P. leptothyrsa* (Gerlach et al., 2010), possibly indicative of the evolutionary relationship between *P. leptothyrsa* and *C. ipecacuanha*.

**Table 1:** Sequence alignment of cyclotides identified from *Carapichea ipecacuanha.*

Cyclotide		Amino acid sequence^†^	Molecular weight (Da)	Identification method^#^	Reference
	Loop	1 2 3 4 5 6			
Caripe 1		GVIP**C**GES**C**VFIP**C**IST-VIG**C**S**C**KNKV**C**YRN	3267.5	MS&tBLASTn	Koehbach and Gruber, 2013; Koehbach et al., 2013a
Caripe 2		G-IP**C**GES**C**VFIP**C**TITALLG**C**S**C**SKKV**C**YKN	3243.4	MS&tBLASTn	Koehbach et al., 2013a
Caripe 3		**X**IP**C**GES**C**VFIP**C**ISAVVGS**C**S**C**–NKV**C**YNN	n.a.^∗^	tBLASTn	Koehbach et al., 2013a
Caripe 4		–LI**C**SST**C**LRIP**C**LSPR–--**C**T**C**RHHI**C**YLN	3030.4	tBLASTn	Koehbach et al., 2013a
Caripe 5		**XC**GES**C**VFIP**C**FTSV–-G**C**S**C**KDKV**C**YRN	n.a.^∗^	tBLASTn	Koehbach et al., 2013a
Caripe 6		GAI-**C**TGT**C**FRNP**C**LSRR–--**C**T**C**RHYI**C**YLN	3199.4	tBLASTn	Koehbach et al., 2013a
Caripe 7		G-IP**C**GES**C**VFIP**C**TVTALLG**C**S**C**KNKV**C**YRN	3253.3	MS&tBLASTn^$^	This study, Wang et al., 2008
Caripe 8		GVIP**C**GES**C**VFIP**C**ITAAI–G**C**S**C**KKKV**C**YRN	3237.4	MS&tBLASTn^$^	This study, Wang et al., 2008
Caripe 9		**XC**VFIP**C**TITALLG**C**S**C**SNNV**C**YKN	n.a.^∗^	tBLASTn^$^	Wang et al., 2008; Koehbach et al., 2013a
Caripe 10		GVIP**C**GES**C**VFIP**C**FSTVI–G**C**S**C**KNKV**C**YRN	3301.3	MS	This study
Caripe 11		GVIP**C**GES**C**VFIP**C**ISTVI–G**C**S**C**KKKV**C**YRN	3281.3	MS	This study
Caripe 12		GVIP**C**GES**C**VFIP**C**FSSVI–G**C**S**C**KNKV**C**YRN	3287.4	MS	This study
Caripe 13		G-IP**C**GES**C**VFIP**C**FTSVF–G**C**S**C**KDKV**C**YRN	3237.2	MS	This study
Psyle E		GVIP**C**GES**C**VFIP**C**ISSVL–G**C**S**C**KNKV**C**YRD	3253.4	MS	This study, Gerlach et al., 2010
	**CYS**	**I II III IV V IV**			

*^†^Isobaric amino acids Leu/Ile were assigned based on chymotrypsin digests (see Supplementary Figures 2–7) and confirmed by amino acid analysis or based on homology to published sequences; ^∗^monoisotopic masses; ^#^the methods that were used for cyclotides characterization were mass spectrometry analysis (MS) and/or transcriptome-mining (tBLASTn); ^$^Cybase (Wang et al., 2008) (www.cybase.org.au) reports the precursor sequences that were identified via BLASTn from the 1 kp-project (Koehbach et al., 2013a) (www.onekp.com); ^∗^not applicable, partial sequences only.*

Sequence analysis of purified *C. ipecacuanha* cyclotides revealed the existence of the conserved six cysteine residues, the conserved glutamic acid (Glu) residue in loop 1, and the absence of the *cis*-Pro residue in loop 5, which classifies them to the bracelet sub-family. The seven characterized cyclotides (caripe 7, 8, 10, 11, 12, 13, psyle E) contain the common GES motif in loop 1, the VFIP motif in loop 2 and a serine residue in loop 4 (**Table 1**). They significantly differ in loops 3 and 5, which are typically recognized to exhibit the highest sequence variability. The only differences in loop 6 are the absence of valine in caripe 7 and caripe 13, and the presence of aspartic acid instead of asparagine in psyle E (**Table 1**). Although caripe 7 and psyle E share the same molecular weight (3254.5 Da), these cyclotides differ in their loop 3 sequences, i.e., TVTALL (caripe 7) vs. ISSVLG (psyle E). Similarly, caripe 8 and 13 (both have the molecular weight 3237.4 Da), contain the sequences ITAAI and FTSVF in loop 3, respectively. The sequence differences of the co-eluting cyclotides of fraction C (caripe10 and 11) and fraction D (caripe 12 and psyle E) (**Figure 4**) can be found in loops 3, 5, and 6. Caripe 10 and 11 differ in only two residues – phenylalanine vs. isoleucine (loop 3) and lysine vs. asparagine (loop 5), respectively – resulting in a molecular weight difference of 20 Da (**Figure 4** and **Table 1**). The other set of co-eluting cyclotides from fraction D, caripe 12 and psyle E, differ by 35 Da, which is due to the presence of phenylalanine, isoleucine (loop 3) and asparagine (loop 6) in caripe 12, instead of isoleucine, leucine (loop 3) and aspartic acid (loop 6) in psyle E. Following the detailed sequence analysis of cyclotides isolated from *C. ipecacuanha* that were able to modulate signaling of the CRF_1_R, it was of interest to characterize this effect in pharmacological detail.

### Pharmacological Characterization of a Novel Cyclotide Antagonist of the CRF_1_ Receptor

**FIGURE 6:**
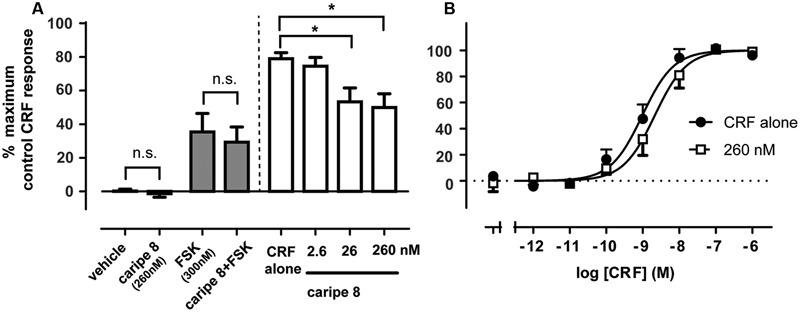
Caripe 8 antagonizes CRF response at the human CRF_1_ receptor. **(A)** Concentration-dependent antagonism of approximate EC_80_ agonist concentrations of CRF by caripe 8. Data are mean ± SEM of three independent experiments performed in triplicate. The asterisk ‘^∗^’ indicates a significant difference of caripe/CRF co-treatment as compared to CRF treatment alone (*p* < 0.05, One-way ANOVA with Dunnett’s *post hoc* test). **(B)** Concentration-dependent stimulation of cAMP accumulation by CRF in the absence (closed circles) and presence of 260 nM caripe 8 (open squares) in HEK293 cells transiently expressing the human CRF_1_R. Data are mean ± SEM of six independent experiments performed in triplicate. LogEC_50_ estimates were significantly different from each other (*p* < 0.05) as determined by *F* test of concentration-response curve data. n.s., non-significant.

We sought to further characterize the inhibitory effect of a purified cyclotide at the CRF_1_R. Based on the previous bioactivity fractionation findings, we selected caripe 8 for subsequent pharmacological analysis as this was the most effective inhibitor of CRF_1_R-mediated luciferase activity (fraction F; **Figure 3C**) relative to the other identified cyclotides. To attempt to disambiguate the effects of cytotoxicity and non-receptor-linked convergent signaling pathways from the previous luciferase bioassay, we sought to examine CRF_1_R-mediated cAMP accumulation. The proximal nature of cAMP generation relative to receptor activation enables more direct assessment of CRF_1_R pharmacological modulation. Similar to the luciferase assay, caripe 8 did not exhibit any intrinsic activity in the cAMP assay. Additionally, no effect by the cyclotide on cAMP accumulation was observed when in combination with submaximal agonist concentrations of forskolin. The initial titration of 2.6, 26, and 260 nM of the cyclotide with an EC_50_ of CRF revealed modest concentration-dependent inhibition of CRF_1_R-mediated cAMP responses (**Figure 6A**), and was observed to reach a threshold (data not shown). Further exploration of this antagonism determined a small (approximately 4.5-fold) reduction of CRF potency by 260 nM caripe 8, but found a lack of effect on the agonist’s maximal response (**Figure 6B**). In contrast, caripe 8 did not inhibit arginine-vasopressin-, or forskolin-stimulated cAMP responses at the vasopressin V_2_-receptor (Supplementary Figure 8), suggesting a CRF_1_R-specific mode-of-action.

## Discussion

Cyclotides have been recently characterized to bind to and activate the oxytocin- and vasopressin V_1a_-receptors, two representative class A GPCRs (Koehbach et al., 2013b). Cyclotides are widely expressed across species of flowering plants (angiosperms) and are particularly abundant in the genus Psychotria *s.l.* of the coffee-family (Rubiaceae). Therefore, in this present study, we sought to expand our knowledge of cyclotides as ligands of other GPCRs. In particular, we identified and analyzed cyclotides from an ipecac root extract – a herbal preparation that was clinically used as an expectorant or an emetic during the past two centuries – that antagonize signaling of the CRF_1_R, a prototypical class B GPCR.

Cyclotides are plant-derived cyclic peptides that are currently of great interest as lead molecules and peptide scaffolds for pharmaceutical drug development (Thell et al., 2016; Craik and Du, 2017). They were first isolated from *O. affinis* (Rubiaceae), a plant used as herbal preparation (‘*kalata-kalata*’) in traditional Congolese medicine for its uterotonic properties (Gran, 1970; Craik et al., 1999; Gran et al., 2000). Evidence-based proof of the plant’s traditional use in childbirth and post-partum care was provided in 2013; Koehbach et al. (2013b) isolated the cyclotide kalata B7 from *O. affinis* and demonstrated it is a partial agonist of the oxytocin- and vasopressin V_1a_-receptors. This cyclotide exhibited only moderate affinity and potency (μM-concentration range), probably due to the larger size of cyclotides as compared to the endogenous neuropeptides, which likely prevents their full penetration of the ligand-binding core of these class A GPCRs (Koehbach and Gruber, 2013). Therefore we rationalized the use of a representative class B GPCR in our pharmacological screening effort, since ligand binding and activation of these receptors involves a more open, accessible and longer N-terminal domain (Hoare, 2005) and therefore, a larger extracellular surface that may better accommodate interaction of the cyclotides with the receptor.

The GPCR superfamily is the largest group of membrane proteins in the human genome, comprising over 800 unique GPCRs distributed throughout the human body (Fredriksson et al., 2003). Of currently available medicines, those targeting GPCRs represent approximately one third of all drugs, despite comprising only 12% of all protein targets (Santos et al., 2017). Given that only a fraction of all human GPCRs are currently targeted, the immense potential for developing new GPCR-based drugs thus remains substantial.

Studying and exploring the potential of plants used in traditional medicine for the discovery of pharmacological lead compounds has been one of the central dogmas of ethnopharmacology and pharmacognosy (Heinrich, 2000). Although the traditional use of ipecac has not been linked to the corticotropin-related disorders, it is important to evaluate any biological effects of other ingredients present in such herbal preparations. Hence we studied the pharmacological activity of cyclotides isolated from a root extract of *C. ipecacuanha* (Rubiaceae) at the CRF_1_R. We utilized a robust luciferase reporter gene assay (Koehbach et al., 2013b) together with cells transiently expressing this receptor. The extract prepared from Carapichea root powder itself had no effect on luciferase induction at those cells, but it inhibited CRF-induced response in a concentration-dependent manner (**Figure 1**). On the other hand, cyclotide extracts derived from other plant species, such as *Viola tricolor* did not result in any marked modulation of CRF ligand-receptor mediated signaling (data not shown), which provided first confidence about the presence of a specific compound(s) in the ipecac extract with the ability to modulate CRF_1_R signaling.

The existence of cyclotides in *C. ipecacuanha* was first reported by Koehbach et al. (2013a). At the time we identified six near-complete sequences of cyclotides (caripe 1 to 6) using a combination of transcriptome-mining and *de novo* peptide sequencing. Caripe cyclotides were identified in leaf as well as root tissue of the plant (Koehbach et al., 2013a). Following our approach to identify novel CRF_1_R-modulating cyclotides, we utilized bioassay-guided fractionation: purified cyclotide fractions were prepared by preparative and semi-preparative HPLC and were screened for pharmacological activity using the luciferase reporter assays, as described above. With the support of MALDI-TOF/TOF MS experiments the cyclotide sequence was characterized by manual *de novo* peptide sequencing. From those cyclotide-positive fractions it was possible to derive seven cyclotide sequences and confirm their existence for the first time at peptide level in Carapichea (summarized in **Table 1**). All cyclotides isolated from *C. ipecacuanha* to date belonged to the bracelet subfamily, which are characterized by the absence of a *cis*-Pro residue in loop 5 (Craik et al., 1999). In agreement with sequencing studies of other bracelet cyclotides, caripe 7, 8, 10–13 and psyle E show highest variability in loops 3 and 5 by the number and types of residues present. Loop 3 contains largely hydrophobic residues apart from a conserved Gly, whereas as loop 5 has a conserved Val/Ile at the C-terminal position. In addition, they all contain at least two positively charged residues in loop 5. Previous structural studies suggested that loop 3 exhibits the only important difference in three-dimensional structures of the different cyclotide subfamilies. In Möbius, exemplified by kalata B1, this four residue loop forms a relatively disordered extended strand, but in bracelet it is sufficiently long to form two turns of a 3^10^-helix (Craik et al., 1999). Therefore it may be speculated that the caripe bracelet cyclotides also contain a helical motif, which may be important for the observed pharmacological activity, since loop 3 is thought to contain the GPCR modulating motif in kalata B7 (Koehbach et al., 2013b).

All purified cyclotide fractions were tested for their potential to inhibit CRF_1_R receptor signaling, and we found that cyclotide fractions B to G had inhibitory potential with varying degrees of apparent effectiveness. Cyclotide fraction F, containing pure caripe 8, was the most effective antagonist, and consequently, caripe 8 was chosen for further detailed pharmacological analysis. Examination of the effects of caripe 8 on cAMP accumulation revealed a much more subtle, yet CRF_1_R-specific, antagonism. This modest and limited antagonism suggests that caripe 8 does not interact competitively with the CRF binding site of the receptor. The greater magnitude of antagonism by caripe 8 in the luciferase assay may be either due to the cyclotide modulating additional cellular pathways (be they CRF_1_R-specific or not) that converge on the (CRE) luciferase output, or an amplification of antagonist effect to this downstream output.

Whether the apparent CRF_1_R antagonizing effects of cyclotides present in a Carapichea root extract have any biological relevance associated with the traditional use of ipecac preparations can only be speculative at this stage. First of all this would require a quantitative analysis of cyclotides in various herbal ipecac preparations, since cyclotide content depends on the method of extraction (Farhadpour et al., 2016) as well as the origin of the plant material and season of harvest (Trabi et al., 2004). Secondly, the pharmacokinetic properties of caripe cyclotides would need to be analyzed. For instance, an ipecac preparation administered orally as an emetic, may result in very low cyclotide uptake, if any, and hence lack of CRF_1_R effects. Lastly, even if cyclotides will be successfully taken up as part of the administration of an ipecac preparation, their CRF_1_R antagonizing effects are mild and likely would not be biologically relevant in the context of traditional use of ipecac. Regardless, the observed receptor-specific nanomolar antagonism provides a novel pharmacophore scaffold to develop new peptide-based antagonists for the CRF_1_R.

Whilst there appears to be no overt sequence or structural homology between the caripe cyclotides, endogenous CRF and other known peptide antagonists (Seidel et al., 2017), unlike previous investigation of kalata B7 (Koehbach et al., 2013b), interrogation of linear segments of these cyclotides may yield lead peptides with enhanced inhibitory activity of CRF_1_R. Without the structural constraints of the cyclotide scaffold, the antagonist pharmacophore may have greater flexibility to interact with the CRF_1_R. Alternatively, considering the structural properties of cyclotides, which exhibit enhanced stability and protease resistance in comparison to linear peptides (Craik et al., 1999; Ireland et al., 2006), our findings of a stable cyclotide ligand of the CRF_1_R could thus be of relevance for future use as a template to design new cyclotide-based GPCR ligands. This has been successfully demonstrated with grafted cyclotide as ligands of the melanocortin 4 receptor (Eliasen et al., 2012). Accordingly, further modifications to the caripe peptides could yield CRF_1_R antagonists with greater pharmacodynamic and improved pharmacokinetic properties. Hence, the cyclotides analyzed during this study from *C. ipecacuanha* could serve as useful tools and templates to develop novel antagonists that target the CRF_1_R.

In conclusion, we identified a cyclotide-containing plant extract that was able to modulate the CRF_1_ receptor which confirms the concept of cyclotides being a treasure trove for drug discovery (Koehbach et al., 2013b). In fact, this is the first time that cyclotides were reported to modulate signaling of class B GPCRs. At a more general level, plants and their active ingredients have always been considered as a rich source for the preparation of herbal medicines and the discovery of novel drugs. There are numerous examples of plant-derived chemicals that have led to the development of important drugs such as Aspirin^®^ and Taxol^®^. Although small molecules, they highlight the potential of cyclotides and other plant-derived peptides might provide for drug discovery and development in the future.

## Materials and Methods

### Peptide Extraction and Enrichment

*Carapichea ipecacuanha* root powder (cat. no. 66804) was purchased from Alfred Galke GmbH (Bad Grund, Germany). The plant material (200 g) was extracted with 1 L of methanol/dichloromethane, 1:1 (v/v) for 18–24 h by maceration under continuous agitation at 25°C. After filtration 0.5 volume of ddH_2_O was added to the extract, and the methanol/water phase, which contained the cyclotides, was obtained by liquid/liquid phase separation. This aqueous mixture was further pre-purified by C_18_ solid-phase extraction. The dried, crude extract was dissolved in 10% methanol/90% ddH_2_O (v/v) and then loaded onto C_18_ material ZEOprep 60 Å, irregular 40–64 μm (Zeochem, Uetikon, Switzerland) that had been activated with methanol and equilibrated with solvent A (100% ddH_2_O/0.1% trifluoroacetic acid, v/v). After washing with 10% solvent B (90% acetonitrile/10% ddH_2_O/0.08% trifluoroacetic acid, v/v/v) it was eluted with 80% solvent B to separate the cyclotide-containing fraction from polar compounds. This pre-purified extract, containing cyclotides, is referred to as ‘ipecac extract.’ Following initial analysis of the pre-purified ipecac extract, cyclotides were enriched by a second cycle of solid phase extraction including washing of the extract with 30% solvent B and elution with 80% solvent B. The resulting fraction is referred to as ‘cyclotide-enriched ipecac fraction.’

### Peptide Fractionation and Purification Using Liquid Chromatography

The dried ipecac extract or cyclotide-enriched ipecac fraction were dissolved in solvent A for liquid chromatography. The mobile phase for all HPLC analysis and purifications consisted of solvent A (100% ddH_2_O/0.1% trifluoroacetic acid, v/v) and solvent B (90% acetonitrile/10% ddH_2_O/0.08% trifluoroacetic acid, v/v/v) and separations were performed on a Dionex 3000 LC unit (Dionex, Amsterdam, The Netherlands), as published earlier (Hellinger et al., 2015a). Analytical RP-HPLC was carried out using a Kromasil C_18_ column (250 mm × 4.6 mm, 5 μm, 100 Å; dichrom GmbH, Marl, Germany) at a flow rate of 1 mL min^-1^, and peptides were separated with linear gradients of solvent B between 15 and 65% at 0.66% min^-1^, including pre- and post-gradient equilibration steps. Cyclotide fractionation and purification was performed at preparative and semi-preparative scale using a Phenomenex Jupiter C_18_ column (250 mm × 21.2 mm, 10 μm, 300 Å; Phenomenex, Aschaffenburg, Germany) and a Kromasil C_18_ column (250 mm × 10 mm, 5 μm, 100 Å). The flow rates were set to 8 and 3 mL min^-1^, respectively, and the cyclotides were separated with linear gradients of solvent B between 15 and 65% at 0.4% min^-1^ (preparative) and 0.57% min^-1^ (semi-preparative), including pre- and post-gradient equilibration steps. Elution profile was monitored via UV absorbance at 214, 254, and 280 nm. Cyclotide fractions were collected manually according to their absorbance 214 nm.

### Peptide Analysis Using MALDI-TOF Mass Spectrometry

Analysis of peptide samples were performed by MS using a MALDI-TOF/TOF 4800 Analyser (AB Sciex, Framingham, MA, United States) operated in reflector positive ion mode acquiring between 2000 and 10000 total shots per spectrum with a laser intensity set between 3200 and 3800. Samples for MS experiments were prepared using α-cyano-hydroxyl-cinnamic acid as matrix (Sigma–Aldrich, St. Louis, MO, United States) in ddH_2_O/acetonitrile/trifluoroacetic acid, 50/50/0.1% (v/v/v) at a concentration of 5 mg mL^-1^. An aliquot of each sample (0.5 μL) was mixed with of matrix solution (3 μL) and spotted directly onto the MALDI target plate. Spectra were processed and analyzed using the Data Explorer Software^TM^ (AB Sciex).

### Reduction, Alkylation, and Enzymatic Digest

Reduction of disulfide bonds was performed by dithiothreitol (Sigma–Aldrich) using 2 μL of a freshly prepared 0.2 M solution (prepared in 0.1 M NH_4_HCO_3_, pH 8.2) that was added to 20 μL of extract, cyclotide-enriched fraction or purified cyclotide dissolved at a concentration of 5 mg mL^-1^ in 0.1 M NH_4_HCO_3_ buffer (pH 8.2). The reduction mixture was allowed to incubate for 30 min at 37°C. Afterward, the reduced samples were carbamidomethylated for 10 min at 23°C in the dark by adding 4 μL of 100 mM iodoacetamide (prepared in 0.1 M NH_4_HCO_3_, pH 8.2 by briefly heating to 65°C for 1 min) to the mixture. The alkylation reaction was stopped by adding 1 μL of 10 mM dithiothreitol. Samples were used for MS analysis or enzymatic digest in preparation for peptide sequencing. For digestion the purified cyclotides (after reduction and alkylation) was performed by adding 2 μL of trypsin, endo-GluC, or chymotrypsin (0.1–0.5 μg μL^-1^, Sigma–Aldrich). All digests were incubated at 37°C for 3–16 h, quenched with concentrated acetic acid (final concentration 3%) and stored at -20°C until further analysis.

### *De Novo* Peptide Sequencing by Tandem Mass Spectrometry

Tandem mass spectra were acquired using a laser energy of 1 kV, with or without the use of collision-induced dissociation. The spectra were examined and sequenced based on assignment of the N-terminal b-ion and C-terminal y-ion series in combination with automated sequence analysis using the Data Explorer Software. The disulfide connectivity of the conserved cysteines (C_I-IV_, C_II-V_, and C_III-V I_) and the isobaric amino acid Leu and Ile were assigned based on homology with published sequences, and/or chymotrypsin fragmentation pattern. In addition the amino acid compositions of novel, purified peptides (caripe 7, 8, and 10; 100–200 μg) were confirmed with high sensitivity amino acid analysis using gas hydrolysis (Australian Proteome Analysis Facility, Sydney, NSW, Australia).

### Cell Culture, Transfection and Functional Luciferase Reporter Assays

HEK293 cells were co-transfected with CRF_1_R plasmid and firefly luciferase containing plasmid pGL4.29 luc2P (Promega GmbH, Mannheim, Germany) expressing the downstream DNA binding elements of GPCR activation (CRE for Gα_s_-coupled signaling). Using CaPO_4_ transfection (Koehbach et al., 2013b), 2.4 × 10^6^ of HEK293 cells per 10 cm dish were prepared and cultured in Dulbecco’s modified Eagle’s medium (PAA, Pasching, Austria) supplemented with 10% fetal bovine serum at 37°C with a humidified atmosphere at 5% CO_2_ for 16 h. Then 6 μL of each needed plasmid (CRF_1_R and CRE for Gα_s_-coupled signaling) with a concentration of 1 μg μL^-1^, was mixed with 287.4 μL H_2_O + CaCl_2_ (250 μL H_2_O + 37.4 μL CaCl_2_) and added to 300 μL of HEPES-buffered saline buffer. After 20 min, the solution was added to the medium and the cells were incubated for 6 h at 37°C. Cells were then seeded into 96-well plates (5 × 10^4^ cells per well) and incubated with different concentrations of cyclotide-enriched fractions with and without 50 nM endogenous agonist CRF in white medium supplemented with 10% fetal calf serum (Gibco, Gaithersburg, MD, United States) plus antibiotics. Following 6 h of incubation at 37°C, medium was removed and then cells were lysed using the luciferase cell culture lysis reagent (Promega GmbH). The luminescence and fluorescence intensities were measured via the Synergy H4 microplate reader (Biotek Instruments, Winooski, VM, United States). The measured luciferase counts were corrected using the fluorescent intensity for the number of cells per well and normalized to the maximum activation of endogenous CRF agonist of the CRF_1_R.

### Peptide Quantification

Molar extinction coefficients (𝜀 at 280 nm) of novel cyclotides were determined according to the formula 𝜀_280_ = nC^∗^120 + nW^∗^5690 + nY^∗^1280 [M^-1^ cm^-1^] (where *n* is the number of residues). The concentration of reconstituted purified peptides was calculated by the Beer-Lambert law based on absorbance measurements at 280 nm using the Nanodrop 1000 (Thermo Fisher Scientific, Waltham, MA, United States). Accordingly the molar extinction coefficient (𝜀_280_) of caripe 8 was calculated to 2000 M^-1^ cm^-1^.

### cAMP Accumulation Assays

Receptor-mediated cAMP accumulation was measured in HEK293 cells transiently transfected with the human CRF_1_R (with C-terminal GFP tag) using the LANCE^®^ Ultra cAMP Detection kit (PerkinElmer, Waltham, MA, United States). To prepare for experiments, HEK293 cells were grown to confluence in a 6-well plate, followed by transfection with 2.5 μg of plasmid DNA per well using the Lipofectamine^®^ 2000 transfection reagent (Thermo Fisher Scientific). After 4–6 h post-transfection, the cell media was replaced, followed by dissociation of cells via trituration and division of the cell suspension to avoid overcrowding over the subsequent 48 h of incubation at 37°C and 5% CO_2_. On the day of assay, cells were dissociated with phosphate buffered saline- (PBS-)EDTA solution (137 mM NaCl, 2.7 mM KCl, 10 mM Na_2_HPO_4_, 1.8 mM KH_2_PO_4_, 1 mM EDTA) and re-suspended in stimulation buffer (1.26 mM CaCl_2_, 0.49 mM MgCl_2_, 0.41 mM MgSO_4_, 5.33 mM KCl, 0.44 mM KH_2_PO_4_, 4.17 mM NaHCO_3_, 138 mM NaCl, 0.34 mM Na_2_HPO_4_, 5.56 mM D-glucose, 5 mM HEPES, 0.5 mM IBMX, 0.1% BSA; pH 7.4). Stimulation of cAMP accumulation via the human CRF_1_R and the human V_2_R used 500 cells and 300 cells (expressing these receptors) per well, respectively, in 384-well white Cellstar^®^ plates (Greiner Bio-One, Kremsmuenster, Austria). Cells were incubated with the respective cognate receptor agonist peptides, in the absence and presence of cyclotide, for 30 min at 23°C in a final volume of 10 μL of stimulation buffer. Termination of cAMP accumulation was performed with the addition of 5 μL of Eu-cAMP tracer solution and 5 μL of ULight-anti-cAMP antibody solution, each prepared in detection buffer, followed by incubation of the plate for 1 h at 23°C with the lid on. After incubation, the plate was read in a Flexstation 3 plate reader (Molecular Devices, Sunnyvale, CA, United States).

### Data Analysis and Statistics

Data from cellular assays were analyzed using GraphPad Prism 7.01 (GraphPad Software, San Diego, CA, United States). Concentration-response curve data were fitted to the three-parameter logistic equation to derive estimates of potency (LogIC_50_, luciferase; LogEC_50_, cAMP) and efficacy (*E*_max_), relative to the maximal agonist control response. CRF LogEC_50_ values in the absence and presence of caripe 8 were statistically compared via extra sum-of-squares *F* test analysis. Other statistical analyses were performed as appropriate using one-way analysis of variance (ANOVA) with Bonferroni’s post-test, and significance taken as *p* < 0.05.

## Author Contributions

CG designed research. MF, PK, CT, and ES performed research. PM and AG contributed new reagents/analytic tools. MF, PK, CT, ES, and CG analyzed data. MF, PK, PM, and CG wrote the paper.

## Conflict of Interest Statement

The authors declare that the research was conducted in the absence of any commercial or financial relationships that could be construed as a potential conflict of interest.
