# Supplementary material for: Cyclotides Isolated from an Ipecac Root Extract Antagonize the Corticotropin Releasing Factor Type 1 Receptor
Source: Front Pharmacol. 2017 Sep 25;8:616. doi: 10.3389/fphar.2017.00616 (PMC5627009; doi:10.3389/fphar.2017.00616)
Supplement: Supplementary file 1 [file Data_Sheet_1.PDF]

Supporting Information

Cyclotides isolated from an ipecac root extract antagonize the corticotropin releasing factor type 1 receptor

Mohsen Fahradpour[#], Peter Keov[#], Carlotta Tognola, Estela Pérez Santamarina, Peter J. McCormick, Alireza Ghassempour and Christian W. Gruber*

[#]These authors contributed equally.

*Correspondence and requests for materials should be addressed to C.W.G. (email: christian.w.gruber@meduniwien.ac.at or c.gruber@uq.edu.au)

Supplementary Figures

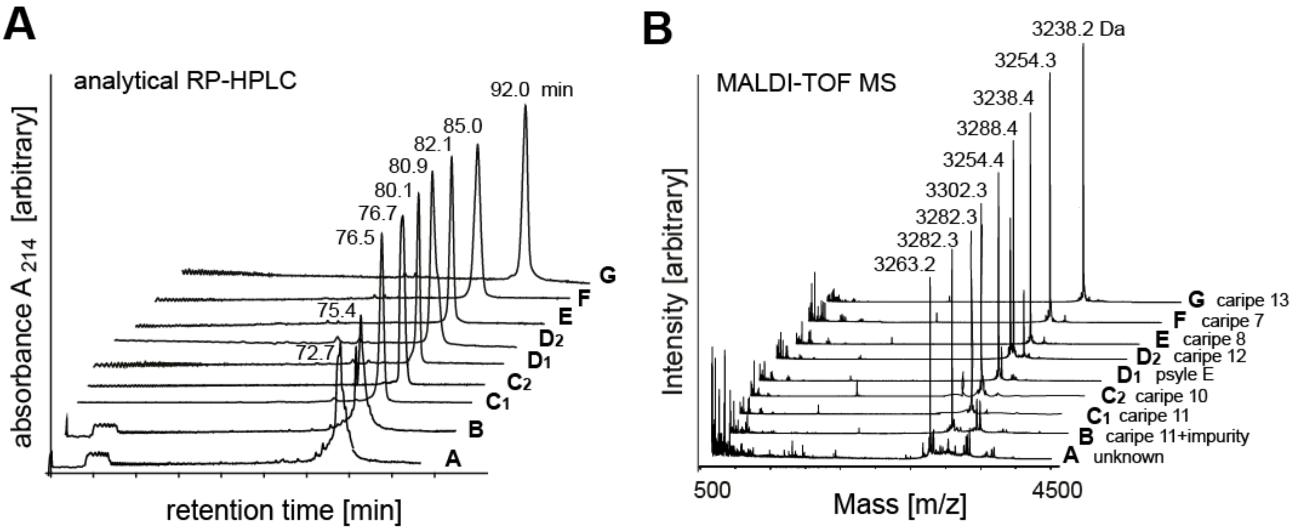

Supplementary Fig. 1. Analysis of cyclotide fractions by HPLC and mass spectrometry. (A) Analytical RP-HPLC chromatograms (A_{214}) of purified cyclotide fractions (A: unknown , B: caripe 11 with impurities, C1: caripe 11, C2: caripe 10, D1: psyle E, D2: caripe 12, E: caripe 8, F: caripe 7 and G: caripe 13) using C_{18} chromatography (Kromasil column; 250 x 4.6 mm, 5 µm, 100 Å) with solvent A (100% H_2O containing 0.1% TFA) and solvent B (10% H_2O/90% acetonitrile/0.08% TFA, v/v/v) as mobile phases. Peptides were eluted with linear gradients of solvent B between 15 and 65% at 0.66% min^{-1}, including pre- and post-gradient equilibration steps. (B) MALDI-TOF MS of purified cyclotide fractions. Masses labelled in the spectra refer to monoisotopic $[M+H]^+$. All fractions are labelled in alphabetical order (A-G) and with cyclotide name.

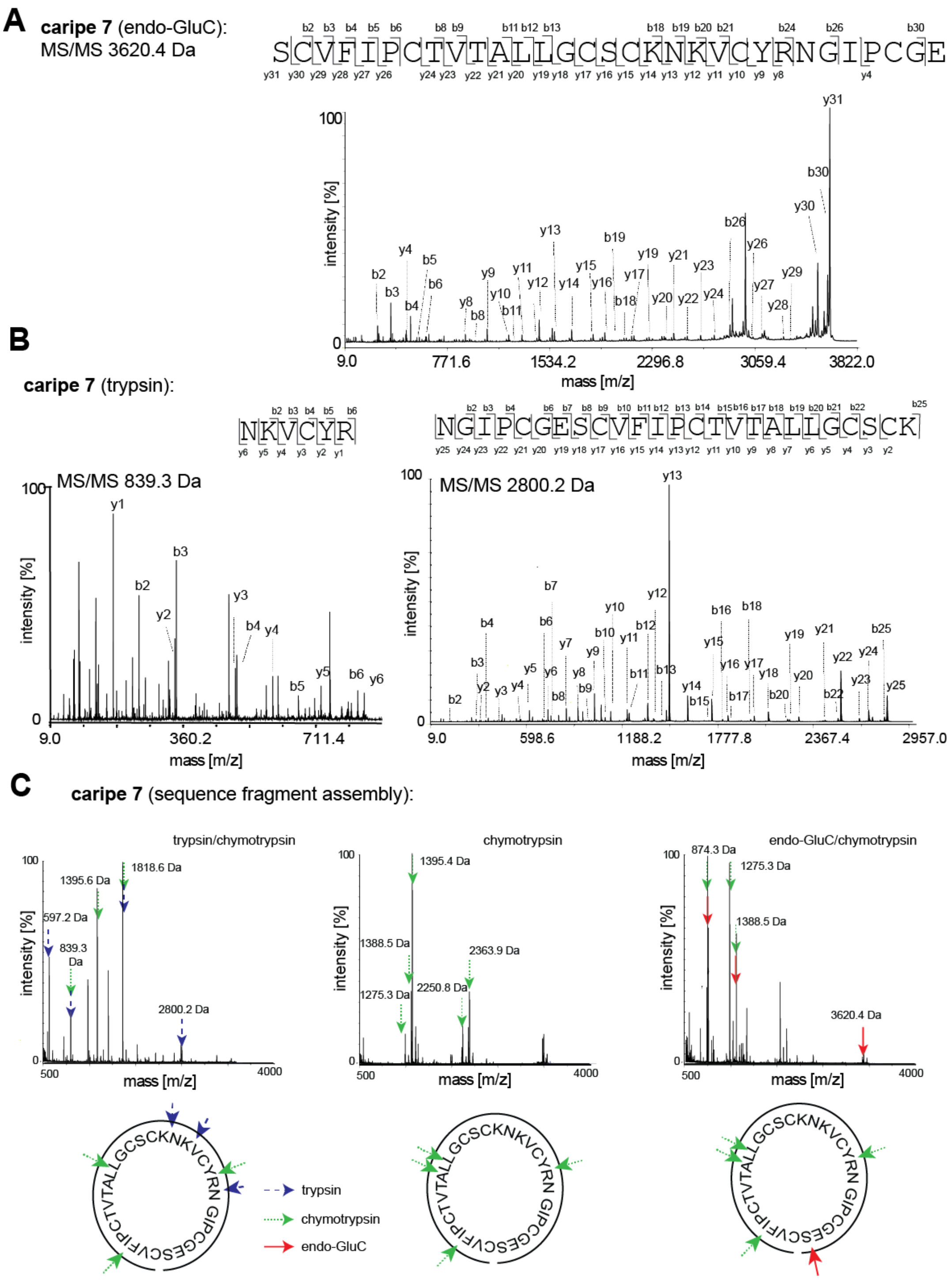

Supplementary Fig. 2. *De novo* sequencing of caripe 7. MS/MS spectra of the (A) precursor with a molecular weight of 3620.4 Da from an endo-GluC digest and the (B) precursors with the molecular weight of 2800.2 Da and 839.3 Da from a tryptic digest are presented. The cyclotide sequences were obtained by assigning the y- and b-ions series as described in the Methods section. (C) To finalize and confirm sequences, a sequence fragment assembly approach was used. MS spectra of three digests using a combination of trypsin and chymotrypsin (lower left), chymotrypsin alone (lower middle), and a combination of endo-GluC and chymotrypsin (lower right) are shown. Fragments of the different enzymes are indicated by arrows (trypsin: blue/dashed line, chymotrypsin: green/dotted line, endo-GluC: red/solid line).

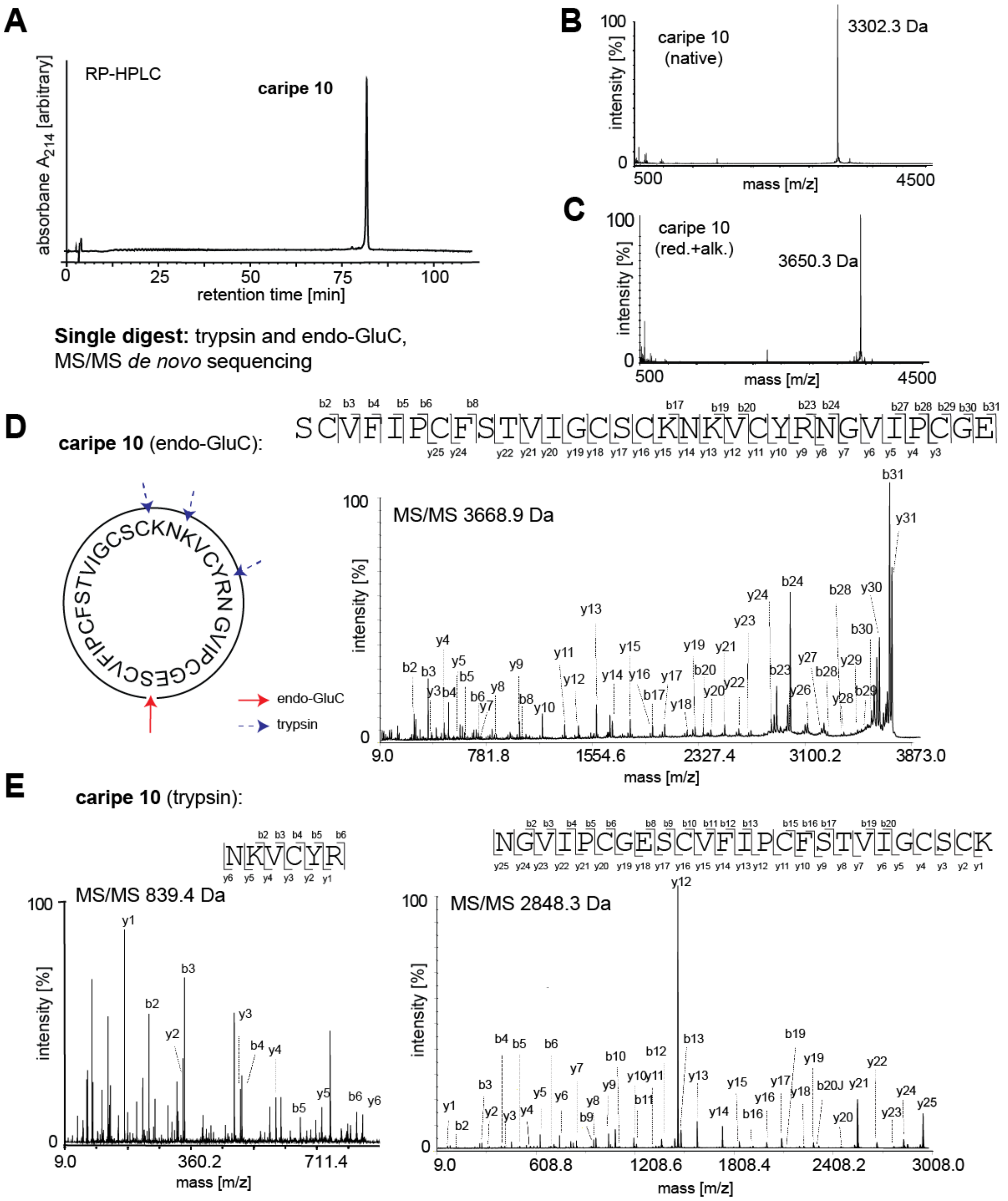

Supplementary Fig. 3. *De novo* sequencing of caripe 10. (A) Purity of the cyclotide was confirmed by analytical RP-HPLC (A_{214} trace). The purified cyclotide (B) with the native molecular weight of 3302.3 Da was characterized by chemical derivatization and MS using sulfhydryl reduction by DTT and iodoacetamide derivatization, which (C) yielded a peptide with the molecular weight of 3650.3 m/z corresponding to S-carbamidomethylation of the six cysteines. *De novo* amino acid sequencing was performed by interpretation of MS/MS fragmentation spectra using (D) endoproteinase Glu C and (E) trypsin digests. The sequence was determined by manual assignment of the N-terminal b-ion and C-terminal y-ion series and the ion fragmentation calculator tool (Data Explorer™, ABSciex).

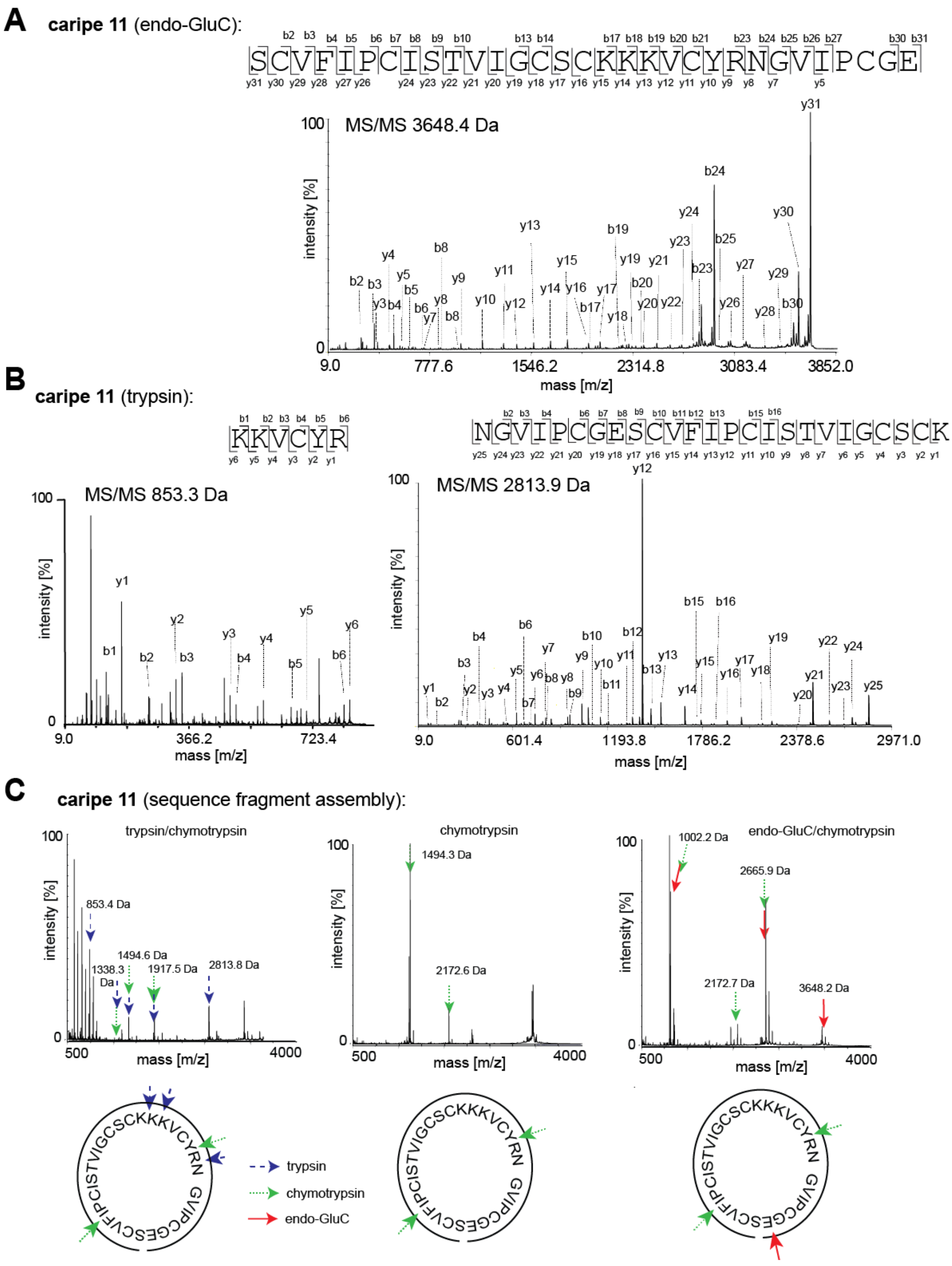

Supplementary Fig. 4. *De novo* sequencing of caripe 11. MS/MS spectra of the (A) precursor with a molecular weight of 3648.4 Da from an endo-GluC digest and the (B) precursors with the molecular weight of 2813.9 Da and 854.3 Da from a tryptic digest are presented. The cyclotide sequences were obtained by assigning the y- and b-ions series as described in the Methods section. (C) To finalize and confirm sequences, a sequence fragment assembly approach was used. MS spectra of three digests using combination of trypsin and chymotrypsin (lower left), chymotrypsin alone (lower middle), and a combination of endo-GluC and chymotrypsin (lower right) are shown. Fragments of the different enzymes are indicated by arrows (trypsin: blue/dashed line, chymotrypsin: green/dotted line, endo-GluC: red/solid line).

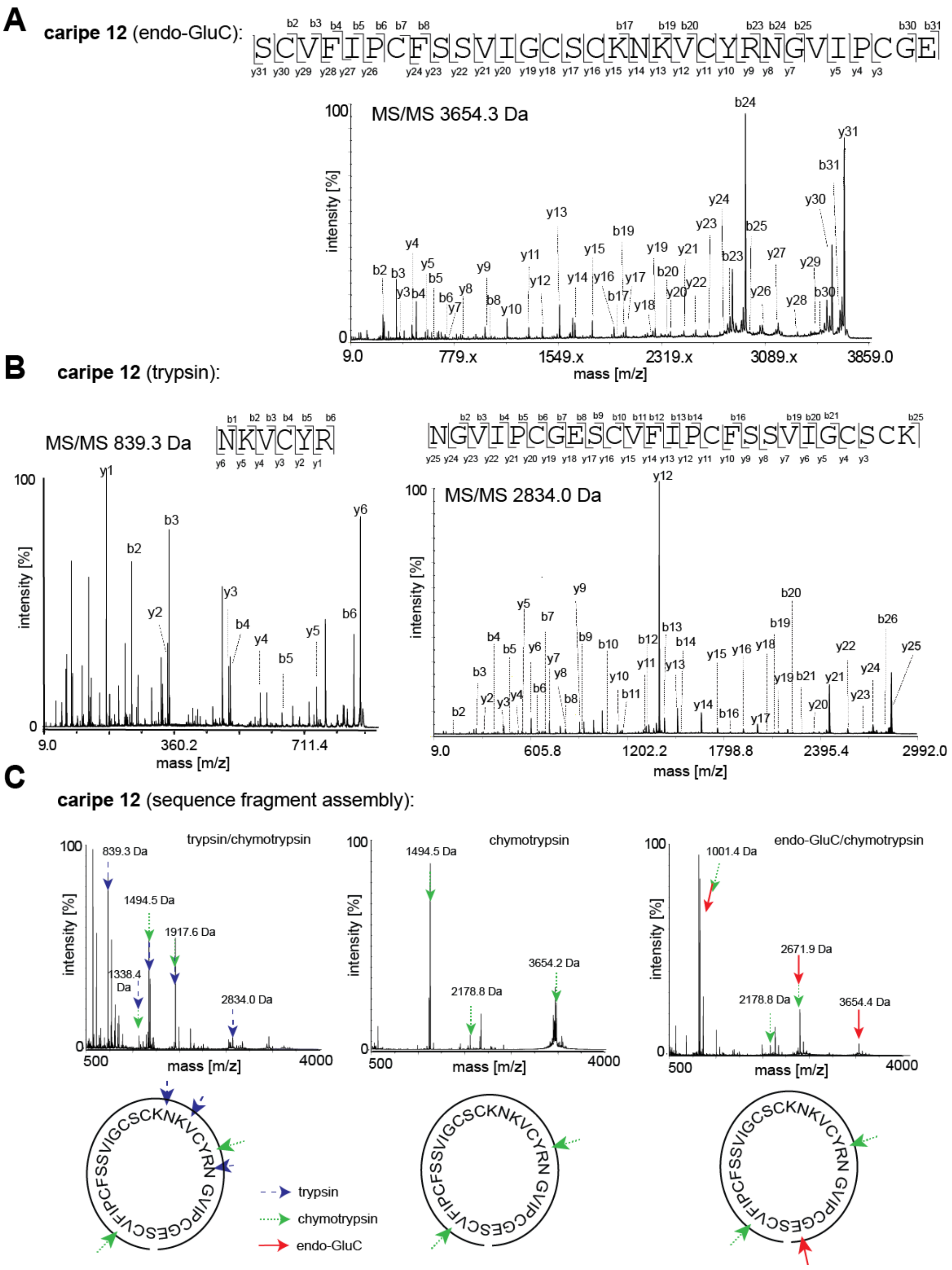

Supplementary Fig. 5. *De novo* sequencing of caripe 12. MS/MS spectra of the (A) precursor with a molecular weight of 3654.3 Da from an endo-GluC digest and the (B) precursors with the molecular weight of 2834.0 Da and 839.3 Da from a tryptic digest are presented. The cyclotide sequences were obtained by assigning the y- and b-ions series as described in the Methods section. (C) To finalize and confirm sequences, a sequence fragment assembly approach was used. MS spectra of three digests using combination of trypsin and chymotrypsin (lower left), chymotrypsin alone (lower middle), and a combination of endo-GluC and chymotrypsin (lower right) are shown. Fragments of the different enzymes are indicated by arrows (trypsin: blue/dashed line, chymotrypsin: green/dotted line, endo-GluC: red/solid line).

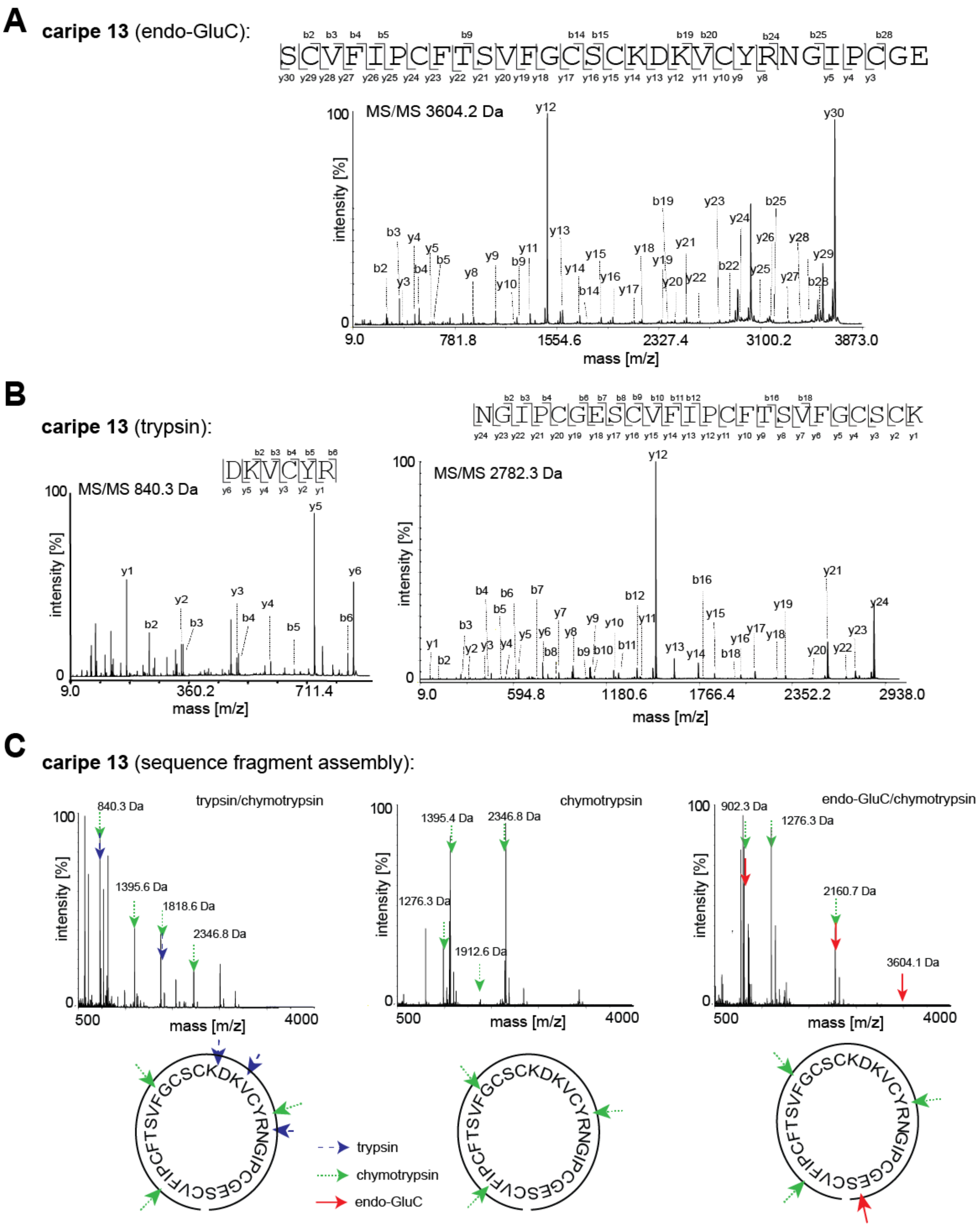

Supplementary Fig. 6. *De novo* sequencing of caripe 13. MS/MS spectra of the (A) precursor with a molecular weight of 3604.24 Da from an endo-GluC digest and the (B) precursors with the molecular weight of 2872.3 Da and 840.3 Da from a tryptic digest are presented. The cyclotide sequences were obtained by assigning the y- and b-ions series as described in the Methods section. (C) To finalize and confirm sequences, a sequence fragment assembly approach was used. MS spectra of three digests using combination of trypsin and chymotrypsin (lower left), chymotrypsin alone (lower middle), and a combination of endo-GluC and chymotrypsin (lower right) are shown. Fragments of the different enzymes are indicated by arrows (trypsin: blue/dashed line, chymotrypsin: green/dotted line, endo-GluC: red/solid line).

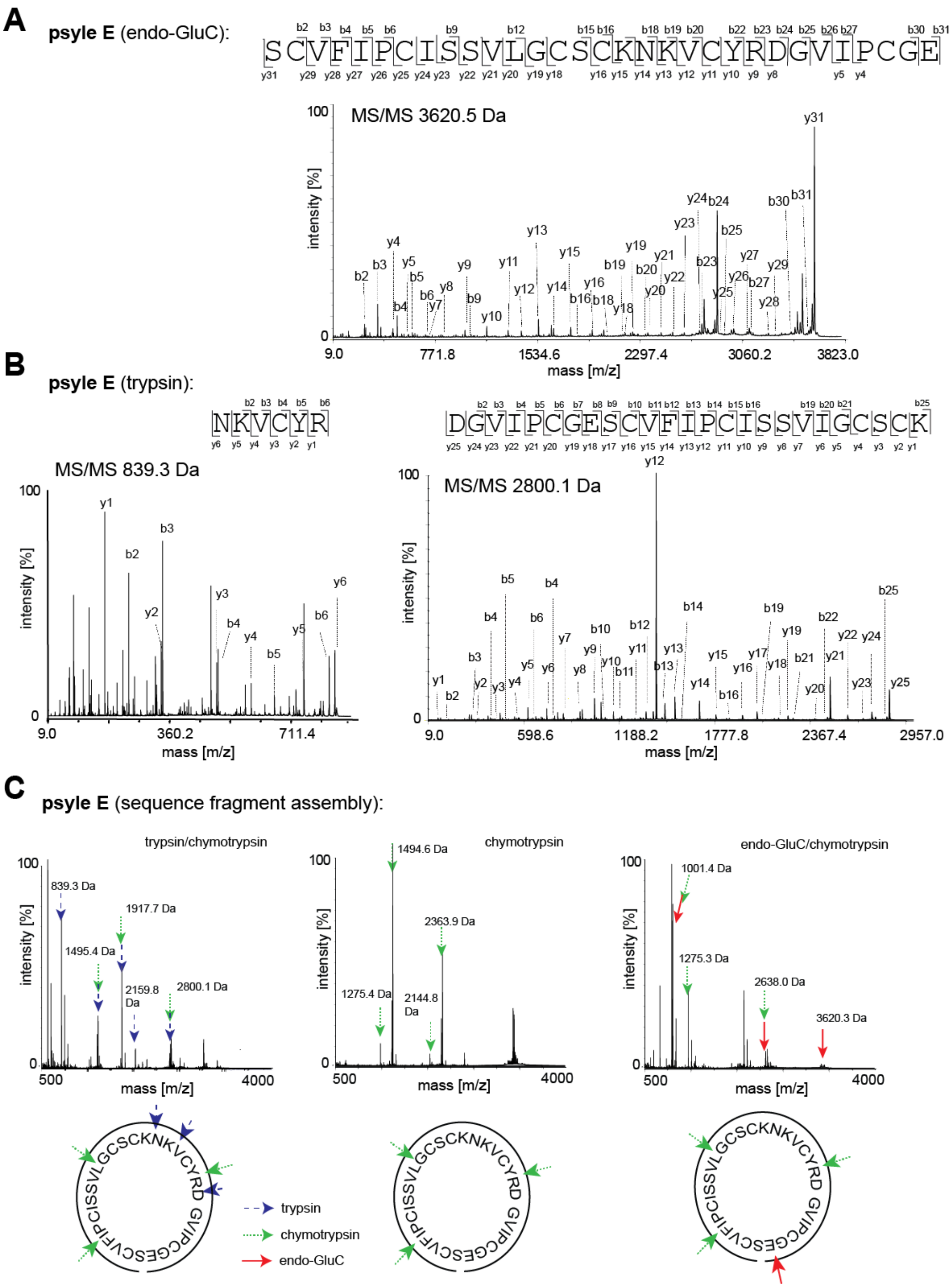

Supplementary Fig. 7. *De novo* sequencing of psyle E. MS/MS spectra of the (A) precursor with a molecular weight of 3620.5 Da from an endo-GluC digest and the (B) precursors with the molecular weight of 2800.1 Da and 839.3 Da from a tryptic digest are presented. The cyclotide sequences were obtained by assigning the y- and b-ions series as described in the Methods section. (C) To finalize and confirm sequences, a sequence fragment assembly approach was used. MS spectra of three digests using combination of trypsin and chymotrypsin (lower left), chymotrypsin alone (lower middle), and a combination of endo-GluC and chymotrypsin (lower right) are shown. Fragments of the different enzymes are indicated by arrows (trypsin: blue/dashed line, chymotrypsin: green/dotted line, endo-GluC: red/solid line).

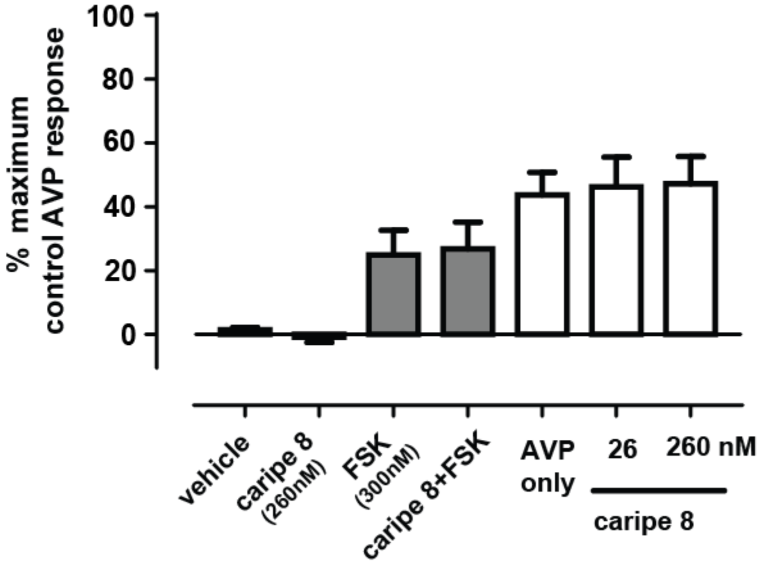

Supplementary Fig. 8. Caripe 8 does not modulate human vasopressin V_2 receptor-mediated cAMP accumulation. Submaximal activation of cAMP accumulation by forskolin (FSK; 300 nM) or arginine-vasopressin (AVP; 50 pM) in HEK293 cells transiently expressing the human V_2-receptor is not altered in the presence of caripe 8 (26 nM & 260 nM). Data are mean ± S.E.M. of five or more independent experiments performed in triplicate. Agonist responses in the presence of caripe 8 were not statistically different to agonist treatments alone (One-way ANOVA).
